# Left main stem disease: percutaneous coronary intervention vs. coronary artery bypass grafting—what about medical management? A case report

**DOI:** 10.1093/ehjcr/ytaf072

**Published:** 2025-02-14

**Authors:** Edyta Kaczmarska-Dyrda, Piotr Nikodem Rudziński, Mariusz Dębski, Zofia Dzielińska, Marcin Demkow

**Affiliations:** Department of Coronary and Structural Heart Diseases, The Cardinal Stefan Wyszynski National Institute of Cardiology, Alpejska 42, 04-628 Warsaw, Poland; Department of Coronary and Structural Heart Diseases, The Cardinal Stefan Wyszynski National Institute of Cardiology, Alpejska 42, 04-628 Warsaw, Poland; Department of Coronary and Structural Heart Diseases, The Cardinal Stefan Wyszynski National Institute of Cardiology, Alpejska 42, 04-628 Warsaw, Poland; Department of Coronary and Structural Heart Diseases, The Cardinal Stefan Wyszynski National Institute of Cardiology, Alpejska 42, 04-628 Warsaw, Poland; Department of Coronary and Structural Heart Diseases, The Cardinal Stefan Wyszynski National Institute of Cardiology, Alpejska 42, 04-628 Warsaw, Poland

**Keywords:** Left main coronary artery stenosis, Bifurcation, Percutaneous coronary intervention, Double kissing crush technique, Case report

## Abstract

**Background:**

Optimal strategy for treating bifurcation lesions in the left main coronary artery (LMCA) remains elusive.

**Case summary:**

We describe a 66-year-old Caucasian male with a risk factor for coronary artery disease, but free of angina, who presented to the hospital after syncope and nsVT diagnosis in the 24 h Holter electrocardiography monitoring. Coronary computed tomography angiography revealed LMCA bifurcation stenosis with concomitant left circumflex artery (LCx) and diagonal branch stenosis. Subsequent coronary angiography confirmed diagnosis. Successful percutaneous coronary intervention (PCI) of bifurcation/distal LMCA disease involving ostium of LAD and LCx has been a challenging procedure.

**Discussion:**

Current guidelines still favour coronary artery bypass grafting (CABG) over PCI for revascularization in LMCA stenosis. However, great progress has been made on PCI; therefore, it has become the compelling alternative procedure to CABG for the LMCA bifurcation stenosis in appropriately selected patients.

**Learning points**

- Advancements in medical devices and interventional techniques have shifted treatment paradigms for LMCA stenosis from CABG to PCI.
- Decision-making should consider factors like anatomical complexity, SYNTAX score, and patient preferences to determine the most suitable revascularization strategy.
- Non-invasive imaging modalities like CCTA play a crucial role in assessing coronary anatomy, plaque burden, and calcifications, aiding in procedural planning and treatment efficacy.

## Introduction

For several decades, coronary artery bypass grafting (CABG) was regarded as the treatment of choice for patients with the left main coronary artery (LMCA) stenosis.^1–3^ However, because of a remarkable advancement of medical devices, antithrombotic therapy, procedural strategies, and interventional cardiologist expertise, percutaneous coronary intervention (PCI) became an attractive option in the treatment of LMCA disease.^4,5^ Technical advances in PCI with a widespread availability of drug-eluting stents led to re-evaluation of the LMCA stenosis treatment and increased the role of PCI. Large registries and randomized clinical trials reported favourable outcomes of PCI in the LMCA stenosis^6,7^; therefore, current guidelines support PCI as a feasible alternative to CABG in selected patients.^8–10^ Concerns about the optimal revascularization strategy for the LMCA stenosis treatment were revised in the long-term clinical trials (SYNTAX, EXCEL) and adopted by the European Society of Cardiology guidelines.^10^ However, location of LMCA stenosis (ostial, mid-shaft, distal/bifurcation), presence of concomitant lesions, diameters of coronary arteries, and the presence of calcifications play an important role in the therapeutic process.

## Summary figure

**Table:** 

Admission and clinical evaluation
66-year-old asymptomatic male
Physical examination: unremarkable
Electrocardiogram: sinus rhythm without ST-segment changes
Echocardiography: preserved left ventricular ejection fraction of 65%
Non-invasive coronary computed tomography angiography (at admission)
60% stenosis of the LMCA bifurcation with minimal lumen area of 5 mm^2^
40% stenosis in the proximal left anterior descending artery (LAD)
60% stenosis in the proximal left circumflex artery (LCx)
Invasive coronary angiography (3 days after admission)
Stenosis of the LMCA bifurcation
Two obstructive stenoses (second diagonal branch and distal LCx)
Decision-making
SYNTAX score calculated: 26
Heart team decides on complex PCI using double kissing (DK) crush bifurcation stenting technique
PCI procedure
Distal LCx and second diagonal branch
LMCA LAD/LCx
Final angiography reveals excellent angiographic result with optimal stent expansion
TIMI 3 flow in all coronary arteries

## Case presentation

**Figure 1:**
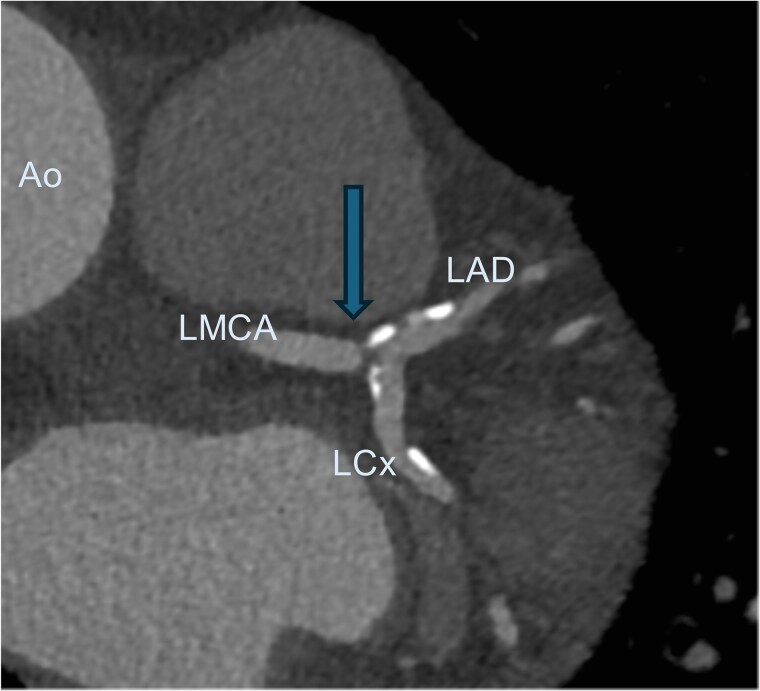
Coronary computed tomography angiography showing left main coronary artery bifurcation stenosis (arrow). Ao, ascending aorta; LAD, left anterior descending artery; LCx, left circumflex artery; LMCA, left main coronary artery.

**Figure 2:**
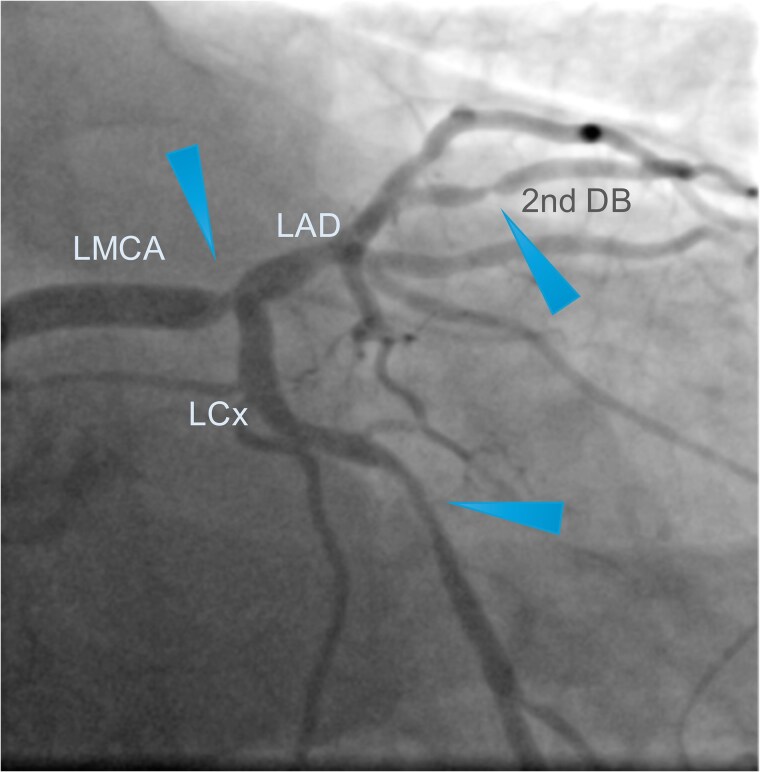
Pre-intervention coronary angiography. Coronary angiography reveals 80% stenosis with filling defect in the bifurcation left main coronary artery. The arrowheads highlight the multivessel character of the coronary artery disease by showing all significant lesions present in the left coronary artery. LAD, left anterior descending artery; LCx, left circumflex artery; LMCA, left main coronary artery; 2nd DB, second diagonal branch.

**Figure 3:**
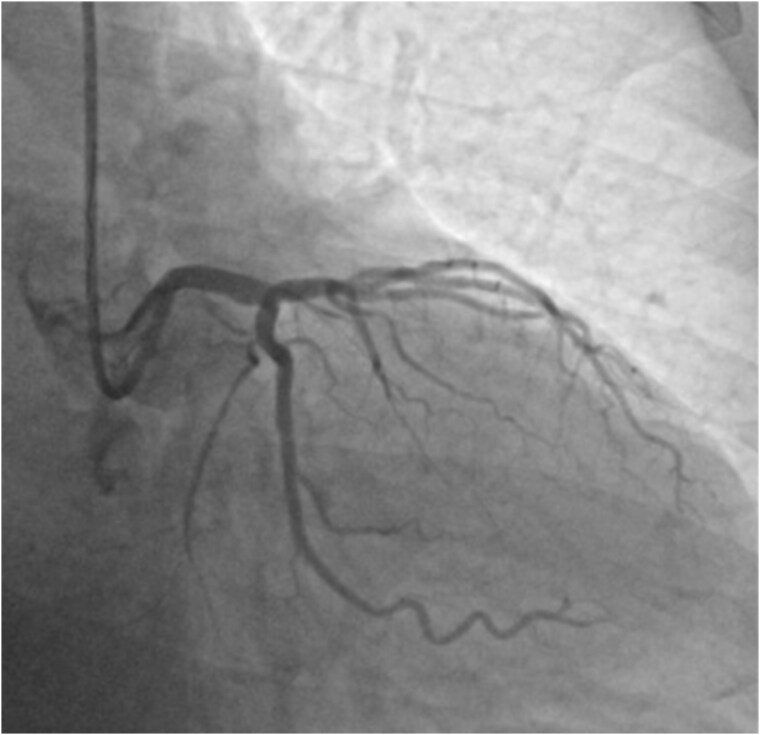
The final coronary angiography with an excellent angiography result, with optimal stent expansion, lesions covered, and TIMI 3 flow in all coronary arteries.

A 66-year-old asymptotic male with hypertension, hyperlipidaemia, and a history of syncope was admitted to the hospital after ambulatory 24 h Holter electrocardiography monitoring, which revealed non-sustained ventricular tachycardia. His physical examination was unremarkable. Electrocardiogram revealed sinus rhythm without any ST-segment changes. 2D echocardiography showed preserved left ventricular ejection fraction of 65%. Non-invasive coronary computed tomography angiography (CCTA) showed 60% stenosis of the LMCA bifurcation with the minimal lumen area of 5 mm^2^, 40% stenosis in the proximal LAD, and 60% stenosis in the proximal LCx (*Figure 1*). Invasive coronary angiography confirmed stenosis of the LMCA bifurcation as well as revealed two obstructive stenoses of the second diagonal branch (DB) and the distal LCx (*Figure 2*). The SYNTAX score was 26, which indicates moderate complexity of coronary artery disease and require careful consideration between PCI and CABG. After the heart team’s (cardiologists, interventional cardiologists, cardiac surgeon) decision and patient’s consent, the complex PCI using DK crush bifurcation stenting technique was planned. Firstly, using the right radial approach, successful PCIs of the distal LCx and the second DB were performed. Secondly, LMCA LAD/LCx procedure was carried out. A 7F (French size) EBU (Extra Back Up, a guiding catheter used to provide maximal support and control during PCI) 3.75 mm guiding catheter was used and two Sion Blue ES (Sion Blue Extra Stiff, a guiding catheter used for high torque control and support) guidewires were placed in the distal segments of LCx and LAD. Then, an Ultimaster Nagomi 3.5/18 mm stent was implanted into the LMCA towards the LCx and partially crushed by a 3.0/15 mm semi-compliant balloon. Subsequently, after rewiring, balloon kissing was performed with a 3.0/15 mm semi-compliant balloon in LCx and a 3.5/15 mm semi-compliant balloon in LAD. Ultimaster Nagomi 4.0/15 mm stent was implanted into the LMCA towards the LAD. Proximal optimization technique (a technique involving inflating a balloon in the proximal part of the larger branch to optimize the opening and ensure better stent apposition and expansion before further stenting of the smaller branch) was performed with a 4.0/8 mm non-compliant balloon and, after rewiring the LCx, re-kissing was performed with a 3.0/15 mm balloon in LCx and a 3.5/15 mm balloon in LAD. Finally, an adequate apposition of LMCA/LCx and LAD stents was confirmed by the intravascular ultrasound monitoring. The final angiography revealed an excellent angiographic result, with optimal stent expansion, lesions covered, and TIMI 3 flow in all coronary arteries (*Figure 3*). The 1-month follow-up of the patient was uneventful.

## Discussion

The optimal revascularization strategy for patients with LMCA disease is still controversial. We present a case of LMCA bifurcation stenosis with concomitant stenosis in LCx, LAD, and DB with SYNTAX score of 26. In such cases, the CABG still remains the standard treatment according to both European Society of Cardiology and American College of Cardiology guidelines.^11,12^ However, the recommendation for PCI depends on anatomical complexity of plaque burden, SYNTAX score, and surgical risk of the patients. According to newly published studies, PCI is a valuable and successful alternative especially among patients with low and intermediate SYNTAX score (≤32). Treatment of LMCA bifurcation stenosis by PCI is still controversial due to distal LMCA stenosis is associated with higher grade lesions (>75%) and higher propensity of associated coronary artery stenosis. Additionally, early and long-term prognosis was worse in patients with LMCA bifurcation stenosis. There are some technical considerations with LMCA bifurcation PCI, which include calcifications, angulation, vessel diameter, concomitant multivessel disease, right coronary artery stenosis, and dominance of Cx. Research shows that while early mortality rates between PCI and CABG may be comparable,^13,14^ CABG is associated with improved long-term outcomes.^14,15^ Specifically, patients undergoing CABG often experience better late survival rates and a reduced incidence of major adverse cardiac and cerebrovascular events compared to those treated with PCI.^16,17^ These findings particularly apply to patients with complex or multivessel coronary artery disease, as demonstrated in several studies, including the SYNTAX and EXCEL trials.^6,7^

In the presented case, CCTA showed anatomy of LMCA stenosis with lack of calcification. Coronary computed tomography angiography has emerged as a powerful non-invasive tool for characterizing the presence, extent, and severity of coronary lesions in patients with stable angina.^18^ During the decision-making process, the heart team should consider the individual characteristics of heart disease and non-cardiac problems, as well as the patient’s preferences, which are sometimes crucial to the decision made. From the patients’ point of view, PCI is less invasive and requires a shorter recovery time than CABG procedure.^19^

## Data Availability

Data supporting the findings of this case report are available from the corresponding author upon reasonable request. Due to privacy concerns, identifying patient information is not publicly accessible. All data shared will be de-identified to ensure confidentiality in accordance with ethical guidelines.

## Lead author biography

Dr Edyta Kaczmarska-Dyrda is an experienced cardiologist and cardiac imaging specialist working in the field of coronary artery disease and structural heart diseases.

**Consent:** The authors confirm that written consent for submission and publication of this case report including the images and associated text has been obtained from the patient in line with COPE guidelines.

**Funding:** None declared.

## References

[1] Chaitman BR, Fisher LD, Bourassa MG, Davis K, Rogers WJ, Maynard C, et al Effect of coronary bypass surgery on survival patterns in subsets of patients with left main coronary artery disease. Report of the Collaborative Study in Coronary Artery Surgery (CASS). Am J Cardiol 1981;48:765–777. 7025604 10.1016/0002-9149(81)90156-9

[2] Takaro T, Peduzzi P, Detre KM, Hultgren HN, Murphy ML, van der Bel-Kahn J, et al Survival in subgroups of patients with left main coronary artery disease. Veterans Administration Cooperative Study of Surgery for Coronary Arterial Occlusive Disease. Circulation 1982;66:14–22. 6979435 10.1161/01.cir.66.1.14

[3] Silber S, Albertsson P, Avilés FF, Camici PG, Colombo A, Hamm C, et al Task Force for Percutaneous Coronary Interventions of the European Society of Cardiology . Guidelines for percutaneous coronary interventions. The Task Force for Percutaneous Coronary Interventions of the European Society of Cardiology. Eur Heart J 2005;26:804–847. 15769784 10.1093/eurheartj/ehi138

[4] Park SJ, Park DW. Percutaneous coronary intervention with stent implantation versus coronary artery bypass surgery for treatment of left main coronary artery disease: is it time to change guidelines? Circ Cardiovasc Interv 2009;2:59–68. 20031694 10.1161/CIRCINTERVENTIONS.108.831701

[5] Park SJ, Ahn JM, Kang SJ. Unprotected left main percutaneous coronary intervention: integrated use of fractional flow reserve and intravascular ultrasound. J Am Heart Assoc 2012;1:e004556. 23316329 10.1161/JAHA.112.004556 PMC3540662

[6] Serruys PW, Morice MC, Kappetein AP, Colombo A, Holmes DR, Mack MJ, et al SYNTAX Investigators . Percutaneous coronary intervention versus coronary-artery bypass grafting for severe coronary artery disease. N Engl J Med 2009;360:961–972. 19228612 10.1056/NEJMoa0804626

[7] Giustino G, Mehran R, Serruys PW, Sabik JF III, Milojevic M, Simonton CA, et al Left main revascularization with PCI or CABG in patients with chronic kidney disease: EXCEL trial. J Am Coll Cardiol 2018;72:754–765. 30092952 10.1016/j.jacc.2018.05.057

[8] Teo KK, Cohen E, Buller C, Hassan A, Carere R, Cox JL, et al Canadian Cardiovascular Society/Canadian Association of Interventional Cardiology/Canadian Society of Cardiac Surgery position statement on revascularization—multivessel coronary artery disease. Can J Cardiol 2014;30:1482–1491. 25475448 10.1016/j.cjca.2014.09.034

[9] Amsterdam EA, Wenger NK, Brindis RG, Casey DE Jr, Ganiats TG, Holmes DR Jr, et al 2014 AHA/ACC guideline for the management of patients with non-ST-elevation acute coronary syndromes: a report of the American College of Cardiology/American Heart Association Task Force on Practice Guidelines. J Am Coll Cardiol 2014;64:e139–e228. 25260718 10.1016/j.jacc.2014.09.017

[10] Neumann FJ, Sousa-Uva M. ‘Ten commandments’ for the 2018 ESC/EACTS Guidelines on Myocardial Revascularization. Eur Heart J 2019;40:79–80. 30615155 10.1093/eurheartj/ehy855

[11] Vrints C, Andreotti F, Koskinas KC, Rossello X, Adamo M, Ainslie J, et al 2024 ESC guidelines for the management of chronic coronary syndromes. Eur Heart J 2024;45:3415–3537. 39210710 10.1093/eurheartj/ehae177

[12] Lawton JS, Tamis-Holland JE, Bangalore S, Bates ER, Beckie TM, Bischoff JM, et al 2021 ACC/AHA/SCAI guideline for coronary artery revascularization: executive summary: a report of the American College of Cardiology/American Heart Association Joint Committee on Clinical Practice Guidelines. Circulation 2022;145:e4–e17. 34882436 10.1161/CIR.0000000000001039

[13] Gaba P, Sabik JF, Murphy SA, Bellavia A, O’Gara PT, Smith PK, et al Percutaneous coronary intervention versus coronary artery bypass grafting in patients with left main disease with and without diabetes: findings from a pooled analysis of 4. Circulation 2024;49:1328–1338. 10.1161/CIRCULATIONAHA.123.065571 38465592

[14] Tam DY, Fang J, Rocha RV, Rao SV, Dzavik V, Lawton J, et al Real-world examination of revascularization strategies for left main coronary disease in Ontario, Canada. J Am Coll Cardiol Intv 2023;16:277–288. 10.1016/j.jcin.2022.10.016 36609048

[15] Persson J, Yan J, Angerås O, Venetsanos D, Jeppsson A, Sjögren I, et al PCI or CABG for left main coronary artery disease: the SWEDEHEART registry. Eur Heart J 2023;44:2833–2842. 37288564 10.1093/eurheartj/ehad369 PMC10406339

[16] Habib RH, Dimitrova KR, Badour SA, Yammine MB, El-Hage-Sleiman AK, Hoffman DM, et al CABG versus PCI: greater benefit in long-term outcomes with multiple arterial bypass grafting. J Am Coll Cardiol 2015;66:1417–1427. 26403338 10.1016/j.jacc.2015.07.060 PMC5473156

[17] Spadaccio C, Benedetto U. Coronary artery bypass grafting (CABG) vs. percutaneous coronary intervention (PCI) in the treatment of multivessel coronary disease: quo vadis?—a review of the evidences on coronary artery disease. Ann Cardiothorac Surg 2018;7:506–515. 30094215 10.21037/acs.2018.05.17 PMC6082779

[18] Tzimas G, Gulsin GS, Takagi H, Mileva N, Sonck J, Muller O, et al Coronary CT angiography to guide percutaneous coronary intervention. Radiol Cardiothorac Imaging 2022;4:e210171. 35782760 10.1148/ryct.210171 PMC8893214

[19] Byrne RA, Fremes S, Capodanno D, Czerny M, Doenst T, Emberson JR, et al 2022 joint ESC/EACTS review of the 2018 guideline recommendations on the revascularization of left main coronary artery disease in patients at low surgical risk and anatomy suitable for PCI or CABG. Eur Heart J 2023;44:4310–4320. 37632756 10.1093/eurheartj/ehad476

